# Plants Used for Tick and Tick-Borne Disease Control in South Africa: Ethnoveterinary Knowledge, Bioactivity Evidence, and Translation Pathways

**DOI:** 10.3390/plants14243720

**Published:** 2025-12-05

**Authors:** Tsireledzo Goodwill Makwarela, Nimmi Seoraj-Pillai, Dikeledi Petunia Malatji, Tshifhiwa Constance Nangammbi

**Affiliations:** 1 Department of Nature Conservation, Faculty of Science, Tshwane University of Technology, Staatsartillerie Rd, Pretoria West, Pretoria 0183, South Africa; seorajpillayn@tut.ac.za (N.S.-P.); nangammbitc@tut.ac.za (T.C.N.); 2 Department of Agriculture and Animal Health, College of Agriculture and Environmental Sciences, University of South Africa, Florida Campus, Roodepoort 1710, South Africa

**Keywords:** acaricide safety, Act 36 of 1947, botanical acaricides, ethnoveterinary medicine, *Lippia javanica*, livestock health, one health, plant-based repellents, South Africa, tick control

## Abstract

Ticks and tick-borne diseases (TBDs) impose a heavy burden on South African livestock systems, particularly in resource-limited communal areas. Conventional acaricides are effective but face rising challenges of resistance, high costs, and concerns for environmental and human health. As a result, there is growing interest in plant-based tick control rooted in ethnoveterinary knowledge. This review examines the landscape of South African ethnoveterinary practices for tick control and assesses the supporting evidence of bioactivity and pathways for translating these remedies into safe, registered products. A narrative review method was applied, drawing on the literature (2000–2025) from databases and local repositories, with emphasis on South African studies documenting plant use against ticks. Communities in Limpopo, Eastern Cape, KwaZulu-Natal, and other provinces utilise a diverse range of botanicals (e.g., *Lippia javanica*, *Tetradenia riparia*, *Clausena anisata*, *Tagetes minuta*, *Melia azedarach*, Eucalyptus spp., *Cymbopogon* spp.) to repel or kill ticks, often through topical applications, fumigation, or livestock housing treatments. Laboratory assays have confirmed acaricidal or repellent activity in many of the cited taxa. For example, *Lippia javanica* and *Tagetes minuta* oils demonstrate strong tick repellency, while extracts of *Tetradenia riparia* and *Calpurnia aurea* exhibit greater than 70% mortality in vitro. Field studies are fewer but promising: a community-led trial with *L. javanica* leaf spray achieved substantial tick reduction (albeit slightly less efficacious than synthetic amitraz). Key gaps include standardisation of plant preparations, safety evaluations (toxicity and residue studies), and alignment with regulatory requirements. Recent regulatory updates in South Africa (Act 36 of 1947) underscore the need for quality, safety, and efficacy data but also create avenues for *low-risk* botanical remedies. Ethnoveterinary plants offer a culturally appropriate and eco-friendly complement to conventional acaricides. Bridging the gap to practical use will require multidisciplinary efforts: validating efficacy in well-designed field trials, ensuring consistency in preparation, assessing safety margins, and navigating registration pathways for plant-based stock remedies. With supportive policy and community engagement, South Africa could pioneer farmer-ready botanical acaricides that mitigate resistance, reduce costs, and advance One Health objectives.

## 1. Introduction

Ticks and the diseases they transmit are a significant threat to livestock health and production in South Africa’s cattle and wildlife systems [1]. Ticks are a substantial source of economic damage to the global livestock industry, with estimated annual costs ranging from USD 20 to 30 billion, primarily due to productivity losses and control measures [2,3]. In South Africa, resource-poor farmers bear a disproportionate burden: tick infestations lead to anaemia, weight loss, hide damage, and deadly TBDs such as heartwater, redwater (babesiosis), and gallsickness (anaplasmosis) [4]. Communal and smallholder farmers rely heavily on government-sponsored dipping programmes and commercial acaricides, yet these face mounting challenges. Widespread acaricide resistance has emerged in multiple tick species, thereby undermining the efficacy of chemical control measures [5,6]. For example, multi-acaricide-resistant populations of the blue tick (*Rhipicephalus microplus*) have been reported in various African regions [6,7,8]. At the same time, the costs of commercial acaricides and limited access to regular dipping make it difficult for small-scale farmers to maintain effective tick control [5,9,10]. Inconsistent dipping services (due to infrastructure or funding issues) further exacerbate tick problems, as noted in surveys where nearly 60% of communal farmers reported gaps in government dipping and had to resort to their own methods [11,12].

These challenges have significant implications for One Health. Improper or excessive use of synthetic acaricides can leave residues in meat and milk, posing food safety risks, and contaminate the environment (soil and water) [13]. Cases of pesticide misuse have raised concerns about human toxicity and environmental pollution [14]. Morpreparation methods (including decoctions and essential oils), and public health concern. Thus, sustainable tick management solutions that are affordable, effective, and environmentally benign are urgently needed. Within this context, ethnoveterinary knowledge, the body of traditional animal healthcare practices, offers promising leads. Across South Africa, indigenous communities have long utilised medicinal plants to protect livestock from ectoparasites [15,16,17]. Ethnoveterinary medicine (EVM) remains prevalent, particularly in areas where access to veterinarians and modern pharmaceuticals is limited [18]. Smallholder farmers often prefer EVM remedies due to their local availability and perceived safety or efficacy [19,20]. In the realm of tick control, numerous plant species have been identified in different provinces as traditional acaricides or repellents. These include aromatic shrubs and trees whose smoke or extracts are believed to deter ticks on animals or in kraals. Recent studies have begun to validate some of these uses under lab conditions, lending scientific credence to traditional practices. Integrating such plant-based methods could enhance tick control in smallholder systems while reducing reliance on expensive chemicals [21].

Early ethnoveterinary research laid a strong historical foundation for botanical tick control studies in Africa. Van Puyvelde et al. [22] conducted one of the first systematic screenings of African plants for acaricidal activity, testing 108 extracts from 42 Rwandan medicinal species and identifying significant tick-killing effects in *Solanum dasyphyllum* fruit and *Neorautanenia mitis* root. These findings highlighted the latent potential of plant-based acaricides as early as the mid-1980s, although traditional practices predated formal research; for instance, farmers in East and West Africa historically used *Nicotiana tabacum* washes, Tephrosia spp., and other local remedies to protect livestock from ticks [23]. By the early 1990s, formal reports emerged from Kenya, where Dipeolu et al. [24] pioneered studies on *Gynandropsis gynandra* (syn. Cleome gynandra). Their study demonstrated in vitro and in vivo anti-tick efficacy, confirming pastoralist observations that cattle treated with crushed Cleome leaves had reduced tick infestations. Subsequent work by Ndungu et al. [25] isolated essential oils from Cleome, showing strong repellency against Rhipicephalus appendiculatus, while Lwande et al. [26] characterised sulfurous and terpenoid compounds responsible for this activity. These foundational studies validated indigenous knowledge and identified plants such as Cleome, Ocimum, Tephrosia, and Neorautanenia as promising sources of acaricidal agents. Concurrent research outside Africa reinforced this concept: Chungsamarnyart et al. [27] in Thailand reported >90% larval mortality in *Boophilus microplus* using local plant extracts, while Indian and Brazilian groups explored neem and tropical weeds for similar effects.

The scope of this review was to examine plants used as tick repellents or acaricides in South Africa, bridging indigenous knowledge and scientific evidence. We focus on botanicals used against ticks (and closely related applications, such as managing tick-infested wounds or maggot infestations), primarily in cattle but also relevant to other livestock. Emphasis is given to South African taxa and ethnoveterinary contexts (e.g., communal dipping tanks, traditional herd management). To structure the evidence pipeline, we review: (i) documentation of ethnoveterinary knowledge (which plants, where, and how they are used), (ii) in vitro bioassays confirming repellency or acaricidal activity of these plants, (iii) in vivo or on-animal studies demonstrating efficacy under field conditions, and (iv) considerations of safety and the regulatory framework (notably South Africa’s Act 36 of 1947 governing stock remedies). This pipeline reflects the translational path from folk remedy to a formally recognised tick control product.

## 2. Methods

This review adopted a narrative synthesis approach to collate ethnoveterinary knowledge and scientific evidence on plants used for tick and tick-borne disease control in South Africa. The objective was to document plant species and traditional preparation methods, evaluate laboratory and field evidence of acaricidal or repellent efficacy, summarise safety and regulatory considerations, and outline pathways for translating folk remedies into registered products. Unlike previous reviews that focused primarily on recent decades, this work deliberately included foundational studies from the mid-1980s onward. The regulatory context was documented with reference to Act 36 of 1947, and the study published in 1985 by Van Puyvelde and colleagues provided seminal acaricidal screening data.

Information sources comprised major scientific databases such as PubMed, Web of Science, and Scopus, as well as Africa-specific platforms like Sabinet and institutional repositories from South African universities. Searches combined geographic, tick-related, and ethnoveterinary keywords using Boolean operators. Representative search strings included combinations such as “(South Africa OR Eastern Cape OR Limpopo OR KwaZulu-Natal OR Mpumalanga OR North West OR Northern Cape OR Western Cape) AND (tick OR Rhipicephalus OR Amblyomma OR Hyalomma OR Haemaphysalis OR Dermacentor) AND (ethnoveterinary OR ethnobotany OR traditional OR plant OR herbal OR essential oil OR extract OR decoction OR repellent)”. Additional searches targeted botanical acaricides and plant-based repellents in livestock contexts, and citation chaining was used to capture older influential works and locally published reports that might not appear in indexed databases.

No lower time limit was imposed during screening to ensure historical continuity. While primary database searches emphasised the literature published between 2010 and 2025, particularly from 2015 onward, reference list mining allowed inclusion of landmark studies from the 1980s and 1990s. Regional studies from neighbouring countries such as Zimbabwe and Tanzania were considered only when the plant species were traditionally used in South Africa, the tick species occurred locally, or the data provided methodological or comparative value. These entries are clearly indicated in the Supplementary Materials.

Eligibility criteria required that sources either documented the use of plants for tick or ectoparasite control in South Africa or evaluated the acaricidal or repellent efficacy of plant materials traditionally used in the country. Ethnographic surveys, laboratory bioassays, field trials, and reviews were included if they were explicitly relevant to ticks or tick-borne diseases. Studies focusing exclusively on internal parasites or human tick-bite treatments were excluded unless they also addressed livestock tick control. Reports lacking sufficient detail to identify plant species, preparation, application, or tick targets were also excluded.

Titles and abstracts were screened for relevance, and full texts were retrieved for potentially eligible records. Duplicates were removed across databases and repositories. Each record was categorised by study type—ethnobotanical survey, laboratory bioassay (including Adult Immersion Test, Larval Packet Test, Larval Immersion Test, and repellency assays), field trial, safety or toxicology assessment, and regulatory guidance—and by geography (South Africa or regional comparator). Where plant identity or tick taxonomy was unclear, species authorities and voucher specimens were cross-checked to resolve ambiguities.

Data extraction captured botanical details such as scientific and common names, plant parts used, and preparation methods, including decoctions and essential oils, along with application techniques and dosage information expressed in standard units (e.g., weight per volume, percentage, milligrams per millilitre). Assay modalities and endpoints were recorded, including mortality rates, repellency measures, LC_50_ and LD_50_ values, and oviposition inhibition. Tick species and life stages were noted, as were safety and toxicity findings, operator protection requirements, and residue considerations for food-producing animals. Regulatory context was documented with reference to Act 36 of 1947 and associated requirements for stock remedy registration.

To ensure comparability across decades, units and symbols were harmonised, assay acronyms standardised, and tick nomenclature aligned with accepted taxonomic conventions. Supplementary Table S1 was updated to consolidate quantitative efficacy metrics such as LC_50_, LC_90_, and LD_50_ values across studies from 1985 to 2025, providing a comprehensive dose–response reference. Where older reports used different units or protocols, values were normalised where possible; otherwise, original units were retained with annotations. Supplementary Table S2 summarises GC–MS chromatographic profiles of key taxa, listing dominant constituents and supporting interpretation of chemotype variation and standardisation requirements.

Although this review did not follow a formal PRISMA protocol, quality checks were applied pragmatically. Ethnobotanical surveys were assessed for clarity of sampling frames and plant identification, laboratory bioassays for adherence to standard protocols and replication, and field trials for design features such as controls and tick burden endpoints. Safety and residue studies were evaluated for species relevance and food-animal implications. Potential sources of bias include publication bias favouring positive outcomes and heterogeneity in protocols across decades. These were mitigated by triangulating ethnoveterinary use reports with experimental evidence and clearly distinguishing strong evidence from anecdotal claims.

Findings are synthesised to trace the translational pipeline from traditional knowledge to scientific validation and regulatory compliance. Results are organised into ethnoveterinary documentation, in vitro and field efficacy (summarised in Table 1 and Supplementary Table S1), phytochemical profiles (Section 4.2 and Supplementary Table S2), and safety and regulatory considerations (Table 2 and Section 5). Limitations include the narrative design and lack of quantitative meta-analysis due to protocol diversity. Nonetheless, inclusion of studies spanning four decades strengthens historical continuity and provides a robust foundation for future research and product development.

## 3. Ethnoveterinary Knowledge Landscape in South Africa

**Figure 1:**
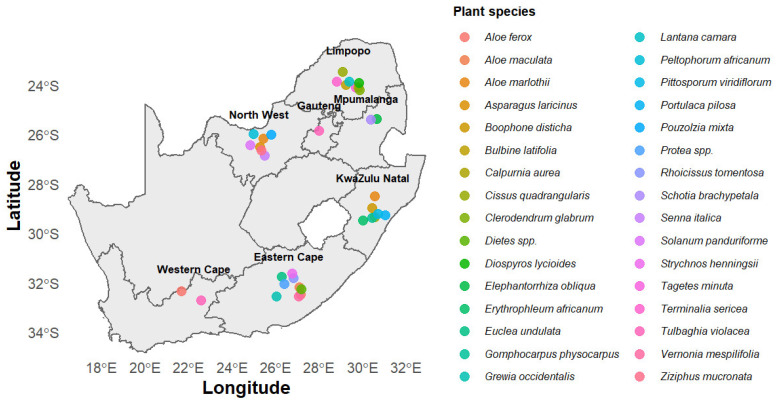
Map of South Africa showing provinces summarising ethnoveterinary plant use against ticks. Data extracted from [20,21,28,41,42,43]. Map created in R version 4.5.1 (2025-06-13 ucrt).

Ethnoveterinary tick control in South Africa is deeply embedded within communal, smallholder, and peri-urban livestock systems, reflecting diverse ecological settings, socio-economic realities, and cultural knowledge networks. Across provinces, farmers adopt a combination of state-led dipping programmes and traditional plant-based remedies, with their choice often determined by resource availability and local practice [10,21,28]. In the Eastern Cape, communal dipping schemes are near-universally attended. Yet, farmers frequently resort to locally sourced adjuncts, most notably *Aloe ferox*, whose leaves are prepared as soaks or sprays to complement dips during shortages or funding constraints [29]. In Limpopo and KwaZulu-Natal, *Lippia javanica* (“zumbani/beukruie”) is the most frequently cited ethnoveterinary plant, prepared as simple aqueous decoctions (10–20% *w*/*v*) and applied as washes or sprays. Field trials confirm its efficacy in reducing tick burdens; however, its performance is inferior to that of synthetic acaricides, such as amitraz-based formulations [30,31]. Other prominent taxa include the exotic *Azadirachta indica* (neem), whose seed oils and kernel extracts are valued for acaricidal properties; *Tagetes minuta* (khakibos), widely applied as an essential-oil repellent; *Clausena anisata* (perdepis), used as spot-on essential oils around the ears and neck; *Ricinus communis* (castor), applied as topical pastes of crushed leaves for engorged ticks; and *Zanthoxylum capense*, cited less consistently but employed in some smallholder settings through crushed stem preparations at infestation sites [32,33,34]. Modes of preparation are pragmatic and community-driven: decoctions of leaves or aerial parts dominate for *L. javanica* and *T. minuta*; essential oils are steam-distilled or purchased for *Tagetes* and *Clausena*, while topical pastes are applied to bite wounds and engorged ticks [35,36]. Application methods range from whole-body washes and hand-sprayed rinses on high-burden areas (such as the neck, brisket, and perineum) to targeted “spot-ons” at the ear bases and tail head. Knowledge transfer is mediated by elder farmers, herders, and herbalists, with occasional support from extension officers who catalogue ethnoveterinary plants or circulate bulletins highlighting promising species, such as provincial guidelines on *L. javanica* [37,38]. Despite their widespread use, ethnoveterinary practices remain unevenly documented and validated. While particular species, including *L. javanica*, *A. indica*, *T. minuta*, *C. anisata*, and *R. communis,* have at least some experimental backing through in vitro assays or limited field trials, others persist primarily as use reports with no systematic biological corroboration. Patterns of reliance also mirror provincial ecology and access (Figure 1): *A. ferox* dominates in the Eastern Cape, *L. javanica* in Limpopo and KwaZulu-Natal, *T. minuta* in Gauteng and Limpopo, neem where commercial products are accessible, and *Clausena* in pockets of KwaZulu-Natal [39,40]. This fragmented yet resilient ethnoveterinary landscape underscores both the adaptive strategies of resource-limited farmers and the critical need for systematic validation, standardisation, and integration of plant-based remedies within sustainable tick management frameworks.

## 4. Experimental Evidence and Bioactive Basis for Efficacy

The validation of ethnoveterinary practices relies on a suite of standardised bioassays that measure the direct and sublethal effects of plant extracts on ticks. Core in vitro methods include the Adult Immersion Test (AIT), which assesses mortality and reproductive impairment in engorged females; the Larval Packet Test (LPT) and Larval Immersion Test (LIT), which evaluate mortality in first-instar larvae; and various repellency assays that measure the ability of a substance to deter tick attachment [44,45,46]. The efficacy observed in these assays is driven by a diverse array of plant secondary metabolites, including terpenoids, phenolics, flavonoids, and alkaloids, which act through neurotoxic, metabolic, and reproductive disruption mechanisms. Detailed quantitative efficacy metrics, including LC_50_ and LC_90_ values for various plant extracts and essential oils tested against tick species, are provided in Supplementary Table S1. These data complement the summary in Table 1 by offering precise dose–response benchmarks for comparative evaluation.

### 4.1. In Vitro and Field Efficacy

South African ethnobotanical taxa have been extensively screened using these bioassays, yielding promising results. As summarised in Table 1, *Lippia javanica* aqueous leaf decoctions (10–20% *w*/*v*) significantly reduced cattle tick burdens in a field trial, demonstrating efficacy that was significant, though slightly inferior to synthetic amitraz [30]. *Azadirachta indica* (neem) seed oil achieved 100% mortality against *Rhipicephalus* (*Boophilus*) *decoloratus* larvae in LPT assays within 27 h at concentrations as low as 20% [47]. *Tagetes minuta* essential oil has demonstrated high repellency, with a low RD_50_ against *R. appendiculatus*, confirming its traditional use as a potent deterrent [40,48]. Extracts from other plants, such as Calpurnia aurea, Cleome gynandra, and Schkuhria pinnata, have also demonstrated high adulticidal activity (greater than 65% mortality) in AITs [49]. Furthermore, *Ricinus communis* leaf extracts have proven effective against organophosphate- and pyrethroid-resistant *R. microplus*, inhibiting oviposition by 36–63% [32,50]. Despite these encouraging laboratory results, field validation remains scarce, with the *L. javanica* trial being a notable exception. This highlights the crucial need for more standardised on-farm studies to confirm efficacy under real-world conditions.

**Table 1:** Experimental evidence of acaricidal and repellent activity of selected South African plants against ticks.

Plant (Family)	Preparation/Extract	Assay	Tick Species/Stage	Doses Tested	Outcome	Refs.
***Lippia javanica*** (Verbenaceae)	Aqueous leaf (10–20% *w*/*v*)	Field spray on cattle	Mixed cattle ticks	10–20%	↓ tick burdens; less effective than amitraz but significant	[30,51]
***Lippia javanica*** (Verbenaceae)	Various leaf extracts	Repellency/Field summary	Mixed species	—	Efficacy near commercial industrial levels (contextual)	
***Azadirachta indica*** (Meliaceae)	Seed kernel water extract (5–10%)	Field (goats/cattle)	*Rhipicephalus* spp.	5–10%	↓ infestation vs. controls; variable efficacy vs. flumethrin/amitraz	[52]
***Azadirachta indica*** (Meliaceae)	Neem seed oil	LPT (larvae)	*R.* (*B.*) *decoloratus* larvae	20–100%	100% mortality within 27 h (at ≥20% concentration)	[47]
***Calpurnia aurea*** (Fabaceae)	Acetone/ethanol leaf/flower	AIT (adults)	*Rhipicephalus turanicus* adults	~1–20%	Adult mortality up to ~81%	[49]
***Cleome gynandra*** (Cleomaceae)	Acetone leaf	AIT (adults)	*R. turanicus* adults	~1–20%	Adult mortality ~78%	[49]
***Tabernaemontana elegans*** (Apocynaceae)	Leaf hydroethanol	AIT (adults)	*R. turanicus* adults	Up to 20%	High adult mortality (≥65% at top dose)	[49]
***Schkuhria pinnata*** (Asteraceae)	Whole-plant extract	AIT (adults)	*R. turanicus* adults	Up to 20%	Adult mortality ~67% (top dose)	[49]
***Aloe rupestris*** (Asphodelaceae)	Leaf extract	AIT (adults)	*R. turanicus* adults	Up to 20%	Adult mortality ~66% (top dose)	[49]
***Tagetes minuta*** (Asteraceae)	Essential oil	Repellency; In vitro	*Rhipicephalus* spp.; *R. appendiculatus*	0.025–1%	High repellency; significant in vitro efficacy	[40,53]
***Cymbopogon citratus*** (Poaceae)	Essential oil; Decoction	LPT; AIT; Repellency	*R. microplus*; *R. sanguineus*	10–40 mg/mL; Topical	Dose-dependent mortality and repellency	[54,55]
***Clausena anisata*** (Rutaceae)	Essential oil	LPT; Topical tests	*A. variegatum*; *R.* (*B.*) spp. (larvae, engorged ♀)	μL/mL; 5–75 μL topical	Low larval LC_50_; impaired oviposition/egg hatch	[33,56]
***Aloe ferox*** (Asphodelaceae)	Powdered crystals (oral)	Field trial (oral)	*R.* (*B.*) *decoloratus*	Label-like dosing	Mixed/insufficient efficacy; palatability issues	[57,58]

**AIT:** Adult Immersion Test. A standard bioassay where adult ticks are immersed in a treatment solution to assess mortality. **LPT:** Larval Packet Test. A standard bioassay where larval ticks are exposed to a treated substrate to determine mortality. **LC_50_:** Lethal Concentration 50. The concentration of a substance required to kill 50% of the test population. **↓:** Decrease or reduction. ***w*/*v*:** Weight per Volume (e.g., 10 g in 100 mL = 10%). **Engorged ♀:** Fully fed adult female ticks. ***R.* (*B.*)*:*** *Rhipicephalus* (*Boophilus*), a typical subgenus of ticks.

### 4.2. Phytochemical Profiles and Bioactivity of Key Species

Most acaricidal plants exhibit distinct chemical profiles that directly shape their bioactivity. *Lippia javanica* presents clear chemotypic variation, with myrcenone-rich, ocimene-rich, and ipsenone-rich profiles, and the strongest activity is consistently linked to monoterpenoid-enriched chemotypes harvested at the pre-flowering stage, when these compounds peak [53,59]. *Azadirachta indica* maintains relatively stable limonoid profiles across environments, with azadirachtin acting through multiple pathways that include feeding deterrence, growth disruption, oviposition inhibition, and contact toxicity, and formulations containing 5 to 10% neem oil reliably achieve complete mortality and reproductive suppression in several tick species [60,61,62]. *Tagetes minuta* contains monoterpene ketones such as tagetone and tagetenone, supported by alpha-terthienyl, and its efficacy depends on chemotypic differences between tagetone-dominated and ocimene-dominated oils, together with phenological transitions from dihydrotagetone-rich pre-flowering leaves to tagetenone-rich post-flowering tissues [63,64]. *Clausena* anisata shows vigorous larvicidal and adulticidal activity due to phenylpropanoids such as trans-anethole and estragole, which induce rapid mortality and marked reproductive failure even at concentrations near 6.25%, making quantification of these markers central to standardisation [65,66]. Comprehensive GC–MS chromatographic profiles of plant extracts and essential oils, including key bioactive compounds, are presented in Supplementary Table S2. These profiles support the interpretation of efficacy trends and standardisation requirements discussed above

## 5. Safety, Toxicity, and Regulatory Considerations

For any ethnoveterinary remedy to transition from traditional use to a registered, marketable product, demonstrating safety is as crucial as proving efficacy. Under South Africa’s Fertilisers, Farm Feeds, Seeds and Remedies Act 36 of 1947, the registration of stock remedies requires scientifically generated dossiers that unequivocally demonstrate quality, safety, and efficacy. This is especially important for plant-based products, where the assumption that “natural” equates to “safe” is a dangerous oversimplification.

### Target Animal and User Safety

The safety profile of a botanical acaricide must be evaluated for the target livestock, the operators handling the product, and the environment. As summarised in Table 2, while many plants show a good safety margin at acaricidal concentrations, critical data gaps persist. For example, *Lippia javanica* spray caused no adverse effects in cattle at concentrations of 10–20% [30]; however, its essential oil requires standard personal protective equipment (PPE) due to potential dermal and ocular irritation. *Azadirachta indica* (neem) is widely used. Yet, high oral doses can cause gastrointestinal upset in livestock, and neem oil handling requires PPE to avoid user exposure [33,67,68]. Essential oils from plants like *Tagetes minuta* and *Clausena anisata*, while effective, have limited livestock safety data and carry risks of dermal irritation or photosensitisation, necessitating careful formulation and usage guidelines [33,69].

**Table 2:** Summary of safety and toxicity considerations for key South African plants used for tick control.

Plant (Family)	Safety in Target Species	Key Mammalian Toxicity Data	Operator and Environmental Safety	Regulatory Status and Remarks	References
*Lippia javanica* (Verbenaceae)	No adverse effects in cattle at 10–20% spray.	Mixed results in mouse studies at high doses; generally tolerable.	Standard PPE is advised for EO due to low environmental persistence.	Widespread community use requires a crucial formulation.	[30]
*Azadirachta indica* (Meliaceae)	Practical: monitor for GI upset with high oral doses.	Neem oil rat LD_50_ ~31.95 g/kg; toxicity in livestock with improper use.	Avoid aquatic release; use PPE for oil handling.	Widely adopted; requires quality standardisation.	[67,68,70]
*Clausena anisata* (Rutaceae)	No livestock field safety data (lab use only).	Mammalian data are sparse; however, they indicate that these species are highly active against ticks.	Potential dermal/eye irritation; standard EO precautions.	Further toxicity and residue studies are needed.	[56,71]
*Tagetes minuta* (Asteraceae)	Dog studies show efficacy; however, there is limited data on livestock.	Can be irritating at high dermal doses; limited LD data.	Risk of dermal irritation and photosensitisation; use PPE.	Best as a repellent adjunct; needs livestock data.	[69]
*Cymbopogon citratus* (Poaceae)	Limited field safety data for tick control.	Generally favourable toxicology at low dermal doses.	EO volatility reduces residue risk; PPE advised.	Promising repellent; requires cattle studies.	[54]
*Calpurnia aurea* (Fabaceae)	No field safety data (lab use only).	Limited data indicate that some related species contain alkaloids.	Unknown residue profile; avoid in food animals.	Prioritise toxicity studies before field use.	[49]
*Schkuhria pinnata* (Asteraceae)	No field safety data (lab use only).	Very sparse mammalian toxicity data.	Unknown operator and environmental risks.	Requires a complete safety assessment.	[49]
*Aloe ferox* (Asphodelaceae)	Poor palatability; practicality concerns.	Known anthraquinone laxative effects: GI distress.	Residue data unclear; avoid in dairy cattle.	Not recommended for tick control.	[57,58]

**GI:** Gastrointestinal. **PPE:** Personal Protective Equipment (e.g., gloves, masks, goggles). **EO:** Essential Oil. **LD_50_:** Lethal Dose 50. The dose required to kill 50% of test subjects.

## 6. Tick Vectors, Small Mammal Hosts, and Ecology

In sub-Saharan Africa, including South Africa, small mammals are important hosts for immature ixodid ticks, sustaining tick populations that later infest livestock and influence farm-level disease risk [72,73,74]. Studies have documented species such as elephant shrews, hedgehogs, and murid rodents hosting larvae and nymphs, creating multi-host networks that shape seasonal and spatial tick abundance in agro-ecological mosaics [73,74]. These hosts maintain tick populations even when cattle are treated, linking wildlife and livestock reservoirs within mixed-use landscapes [72,73]. Beyond vector ecology, small mammals may indirectly affect pathogen cycles, including Theileria spp., by sustaining tick vectors in grazing systems with wildlife–livestock interfaces [75,76,77]. Pathogen profiling in ixodid ticks from the Eastern Cape further underscores the role of vertebrate hosts, including small mammals, in shaping pathogen dynamics such as circulating piroplasms and Theileria-like agents [75]. Collectively, evidence from South Africa and broader African contexts supports a One Health perspective that recognises interconnected wildlife, livestock, and environmental factors in tick-borne disease systems [77]. Future research should integrate longitudinal field data and molecular surveillance to quantify the contribution of small mammals to tick ecology and pathogen transmission, reinforcing the need for integrated tick control strategies beyond livestock treatment [73,74,75].

## 7. Cost and Accessibility of Ethnoveterinary Acaricides Compared with Commercial Synthetics

Ethnoveterinary plant-based acaricides offer high cost and accessibility advantages for South African smallholder farmers compared with commercial synthetic products. Conventional acaricides remain effective but impose high financial burdens, with Amitraz priced at R 347 to R 569 per litre, pour-on formulations ranging from R 284 to more than R 4 039, and large-scale dips exceeding R 6 212, resulting in annual per-animal treatment costs of R 9.29 to R 126.86 [78]. These pressures are intensified by supply disruptions and resistance linked to improper use [3,79]. In contrast, communities often rely on locally available plants such as *Lippia javanica*, *Azadirachta indica*, *Maerua edulis*, *Tagetes minuta*, *Aloe ferox*, and *Aloe marlothii*, which can be harvested at minimal cash cost, with labour being the primary input [80,81]. Empirical studies report that *Lippia javanica* water extracts and 2% neem seed preparations can achieve control comparable to that of standard pyrethroids. At the same time, *Maerua edulis* formulations have matched Amitraz efficacy under field conditions [82,83,84]. Although plant-based acaricides require time for preparation and show variable efficacy, they bypass transport and retail markups that restrict access to commercial products in remote areas. At the development stage, botanical pesticides cost approximately USD 3 million to USD 7 million and take about 3 years to reach the market, compared with USD 250 million to USD 400 million and 8 to 10 years for synthetic chemicals [85]. Integrating validated plant-based treatments with the prudent use of commercial acaricides or vaccines, such as Bm eighty-six, offers a more economically sustainable and context-appropriate strategy for tick control in South Africa [86,87].

## 8. Residue and Environmental Considerations

When plant-based acaricides are applied to food-producing animals, residue depletion becomes a paramount concern. While many essential oil constituents are rapidly metabolised, lipophilic compounds, such as terpenoids, can accumulate in fat depots [88]. In the absence of comprehensive residue depletion studies, regulators often mandate conservative withdrawal periods for meat and milk to safeguard consumer health and comply with international trade standards [89]. Environmental safety must also be considered; for instance, neem oil should be kept out of aquatic systems. The high volatility of some essential oils, while reducing residue risk, requires an assessment of inhalation exposure for operators [90].

### Sustainability Considerations for Ethnoveterinary Acaricides

Sustainable ethnoveterinary tick control depends on the effectiveness of plant-based acaricides and their alignment with conservation, cultivation potential, and local knowledge systems. The literature emphasises that wild harvesting must be balanced with deliberate cultivation and ecological risk assessment to avoid depletion of vulnerable taxa [23]. Early findings on *Neorautanenia mitis* root extracts illustrated how high acaricidal demand can pressure wild populations without cultivation efforts [22]. Surveys across eastern and southern Africa show frequent use of *Lippia javanica*, *Tagetes minuta*, and *Aloe* species, with rising concerns over sustainable harvesting thresholds [91]. Cultivation studies confirm that *Lippia javanica*, *Tagetes minuta*, and *Azadirachta indica* can be integrated into small-scale farming systems to supply renewable biomass and minimise wild extraction [30,92]. Initiatives such as ADAPPT offer farmer-led models for propagation, training, and seed distribution [93]. Botanical pesticides also present environmental advantages through faster degradation and narrower toxicity spectra, although recent reviews recommend careful non-target risk assessment [94,95]. Finally, ethnoveterinary knowledge and plant resources remain vulnerable to overuse and cultural erosion, highlighting the need for documentation, community-based conservation, and integration of traditional practices with formal veterinary and extension platforms [92,96].

## 9. The Regulatory Pathway for Botanicals in South Africa

Navigating Act 36 of 1947 is the definitive pathway to market for a plant-based stock remedy, a process that requires the compilation of a rigorous, comprehensive dossier. This dossier must include a standardised formulation with clearly defined botanical sources, extraction methods, and chemical profiles, supported by marker compounds to ensure batch-to-batch consistency. It further requires robust efficacy data derived from standardised bioassays, such as the Adult Immersion Test and Larval Packet Test, supported by at least one controlled field trial demonstrating significant tick reduction. Comprehensive safety studies are essential and should encompass Target Animal Safety assessments at intended and elevated doses, including evaluations of dermal and ocular tolerance, as well as operator exposure risk assessments. For food animals, residue depletion studies are needed to establish scientifically justified withdrawal periods for meat and milk. Finally, the dossier is completed with product stability data and transparent, compliant labelling. Bridging the rich ethnoveterinary knowledge base to compliant products, therefore, depends on a dual focus: the methodological harmonisation of bioassays to yield comparable data and the generation of regulatory-grade safety and residue studies. Only through this comprehensive and evidence-based approach can promising plant taxa be successfully advanced into marketable tools for integrated tick management.

## 10. Knowledge Gaps and Future Research Priorities

Several interdisciplinary research gaps hinder the translation of ethnoveterinary knowledge into practical tick control tools. First and foremost, there is a need for standardised bioassays and formulations. The heterogeneity in extraction solvents, bioassay protocols, and tick life stages tested makes meta-analysis and product benchmarking difficult [97]. Research must prioritise the development and adoption of standardised methods, potentially including emerging tools such as the Resistance Intensity Test (RIT), for evaluating both synthetic and botanical acaricides [46]. A critical bottleneck is the scarcity of field trials. While laboratory results are promising, robust, replicated on-farm studies assessing tick load reduction, animal productivity metrics, and cost-effectiveness are rare [98]. Future work must validate laboratory findings in real-world settings by exploring integration strategies, such as weekly botanical repellents between conventional dipping cycles.

Furthermore, comprehensive safety and residue profiles for the most promising plants are lacking. Priority should be given to generating regulatory-grade Target Animal Safety (TAS) data and residue depletion curves for key species, such as *L. javanica*, *T. minuta*, and *C. anisata*, to facilitate registration. Finally, innovative research should explore multi-herb synergies and novel delivery systems. Combining plant extracts may enhance efficacy and delay the development of resistance [99]. Investigating nanoformulations and slow-release systems could also improve product performance, stability, and user compliance [100]. Addressing these gaps through collaborative, multidisciplinary research is essential for developing effective, safe, and commercially viable botanical acaricides for South African farmers.

## 11. Conclusions and Recommendations

The significant burden imposed by ticks and tick-borne diseases on South African livestock, particularly in resource-limited communal farming systems, underscores the urgent need for sustainable, accessible control strategies. This review confirms that ethnoveterinary knowledge offers a rich and culturally relevant repository of potential solutions, utilising a diverse array of botanicals, including *Lippia javanica*, *Azadirachta indica*, and *Tagetes minuta*, for this purpose. A growing body of scientific evidence now validates these traditional practices, with in vitro bioassays consistently demonstrating the potent acaricidal and repellent properties of these plants and identifying key bioactive constituents, including terpenoids, phenolics, and alkaloids, that underpin their efficacy.

Despite this promising foundation, a substantial translational gap remains between traditional use and the availability of registered, standardised products. The scarcity of robust field trials, coupled with a lack of comprehensive safety evaluations and residue data, represents the primary bottleneck. The South African regulatory framework, governed by the Fertilisers, Farm Feeds, Seeds and Remedies Act (Act 36 of 1947), while rigorous, provides a clear, science-based pathway for registering plant-based stock remedies, mandating demonstrable quality, safety, and efficacy.

To bridge this gap and harness the full potential of ethnoveterinary plants within integrated tick management programmes, a concerted, multidisciplinary effort is required. Future research must prioritise field validation through well-controlled trials that assess not only tick reduction but also animal productivity and economic viability. Concurrently, there is a critical need to standardise bioassay methodologies and develop stable, farmer-friendly formulations from well-characterised plant materials. Generating regulatory-grade data on target animal safety and residue depletion for the most promising candidate species is a crucial step in compiling compliant registration dossiers. Furthermore, exploring synergistic effects in multi-plant formulations could enhance efficacy and delay the development of resistance.

For policymakers and regulatory bodies, developing specific guidelines for these “low-risk” botanicals within the existing Act 36 framework would provide much-needed clarity and streamline the development process. Fostering collaborative platforms that unite traditional knowledge holders, researchers, and commercial partners is crucial for driving innovation. For extension services and farmers, promoting best practices for the safe and effective preparation of traditional remedies remains a vital interim measure, alongside efforts to document and preserve this invaluable knowledge. In conclusion, by championing a translational pathway that respects indigenous expertise while adhering to scientific and regulatory rigour, South Africa can pioneer the development of effective, farmer-ready botanical acaricides. Such innovations promise to mitigate acaricide resistance, reduce economic burdens on smallholder farmers, and advance the integrated, sustainable management of livestock health in line with One Health principles.

## Data Availability

Data are provided within the body of the article.

## Supplementary Materials

The following supporting information can be downloaded at https://www.mdpi.com/article/10.3390/plants14243720/s1, Supplementary Table S1: Updated quantitative efficacy metrics (LC_50_, LC_90_, LD_50_) for plant extracts and essential oils tested against tick species (1985–2025). Supplementary Table S2: GC–MS chromatographic analysis of plant extracts and essential oils used in ethnoveterinary tick control, including key bioactive compounds and references. References [54,101,102,103,104,105,106,107,108,109,110,111,112,113,114,115,116,117,118,119,120,121,122,123,124,125,126,127,128,129,130,131,132,133,134,135,136,137,138,139,140,141,142,143,144,145,146,147] are cited in the supplementary materials.

## Abbreviations

%	Percentage
↑	Increase
↓	Decrease or reduction
♀	Female (engorged adult tick stage)
*A. sculptum*	*Amblyomma sculptum*
*A. variegatum*	*Amblyomma variegatum*
Act 36 of 1947	Fertilisers, Farm Feeds, Seeds and Remedies Act (South Africa)
ADMET	Absorption, Distribution, Metabolism, Excretion and Toxicity
AI	Artificial Intelligence
AIT	Adult Immersion Test
Anaplasma spp.	Anaplasma species
Babesia spp.	Babesia species
Bm86	Boophilus microplus antigen used in anti-tick vaccines
CFU	Colony Forming Unit
*D. nitens*	*Dermacentor nitens*
DNA	Deoxyribonucleic Acid
EO	Essential Oil
EOs	Essential Oils (plural)
EVM	Ethnoveterinary Medicine
GC–MS	Gas Chromatography–Mass Spectrometry
GI	Gastrointestinal
GIS	Geographic Information Systems
*H. anatolicum*	*Hyalomma anatolicum*
*H. longicornis*	*Haemaphysalis longicornis*
*H. rufipes*	*Hyalomma rufipes*
*H. scupense*	*Hyalomma scupense*
LC50	Lethal Concentration 50
LD50	Lethal Dose 50
LIT	Larval Immersion Test
LPT	Larval Packet Test
mg/mL	Milligram per millilitre
NMR	Nuclear Magnetic Resonance
PPE	Personal Protective Equipment
R. (B.)	Rhipicephalus (Boophilus) subgenus designation
*R.* (*B.*) *decoloratus*	*Rhipicephalus* (*Boophilus*) *decoloratus*
*R. appendiculatus*	*Rhipicephalus appendiculatus*
*R. microplus*	*Rhipicephalus microplus*
*R. sanguineus*	*Rhipicephalus sanguineus*
*R. turanicus*	*Rhipicephalus turanicus*
RD50	Repellent Dose 50
RIT	Resistance Intensity Test
spp.	Species plural (multiple species within a genus)
TAS	Target Animal Safety
TBD	Tick-Borne Disease
Theileria spp.	Theileria species
USD	United States Dollar
*w*/*v*	Weight per Volume
μL	Microlitre
