# Supplementary material for: Plants Used for Tick and Tick-Borne Disease Control in South Africa: Ethnoveterinary Knowledge, Bioactivity Evidence, and Translation Pathways
Source: Plants (Basel). 2025 Dec 5;14(24):3720. doi: 10.3390/plants14243720 (PMC12737041; doi:10.3390/plants14243720)
Supplement: Supplementary file 1 [file plants-14-03720-s001.zip › plants-4017000-supplementary.pdf]

plants

MDPI

Supplementary Table S1. Quantitative efficacy metrics (LC_{50}, LC_{90}, LD_{50}) for plant extracts and essential oils tested against tick species.

Plant extracts or oils	Tick species	Quantitative LC_{50} values	Reference
Carthamus tinctorius; *Nasturtium officinale*; mixture 1:1	*Hyalomma scupense*	LC_{50} 61.78 mg/mL; LC_{50} 84.16 mg/mL; LC_{50} 47.96 mg/mL	[1]
Origanum onites	*Rhipicephalus turanicus*	LC_{50} 2.34 %; LC90 7.12 %	[2]
Alstonia scholaris; *Sida cordifolia*	*Hyalomma anatolicum*	LC_{50} 0.71 %; LC_{50} 0.42 %	[3]
Thymol; 1.8 cineole	*Rhipicephalus microplus*	Thymol LC_{50} 0.28 mg/mL and LC90 0.64 mg/mL; cineole LC_{50} 0.64 mg/mL and LC90 2.66 mg/mL	[4]
Ocimum gratissimum; *Alpinia zerumbet*; *Mesosphaerum suaveolens*	*Rhipicephalus microplus*	LC_{50} 11.9 mg/mL; LC_{50} 19.7 mg/mL; LC_{50} 51.6 mg/mL	[5]
O. minutiflorum oil; *D. hastata* oil	*Rhipicephalus sanguineus*	*O. minutiflorum* LC_{50} 0.101 % and LC90 0.125 %; *D. hastata* LC_{50} 0.937 % and LC90 2.1 %	[6]
Cinnamomum cassia extract; *C. cassia* oil; (E) cinnamaldehyde	*Haemaphysalis longicornis* larvae and nymphs	Extract LC_{50} 11.56 mg/mL and LC_{50} 49.18 mg/mL; oil LC_{50} 3.81 mg/mL and LC_{50} 21.31 mg/mL; cinnamaldehyde LC_{50} 3.15 mg/mL and LC_{50} 16.93 mg/mL	[7]
A. columnaris oil; *N. pancheri* oil	*Rhipicephalus microplus*	Effective oil equivalents 2.36 and 3.51 (LC_{50} equivalent)	[8]
C. sempervirens ethanol extract	*Rhipicephalus annulatus*	LC_{50} 12.2 %	[9]
Thymol	*Rhipicephalus microplus*	LC_{50} 3.45 mg/mL	[10]
Ocotea elegans oil	*Rhipicephalus microplus*	LC_{50} 59.68 mg/mL	[11]
Acmella oleracea hexane extract	*Rhipicephalus microplus*	LC_{50} 79.7 mg/mL	[12]
(E) cinnamaldehyde; thymol; carvacrol	*Amblyomma sculptum*; *Dermacentor nitens*	For *A. sculptum*: LC_{50} 1.40 mg/mL, LC_{50} 2.04 mg/mL, LC_{50} 3.49 mg/mL. For *D. nitens*: LC_{50} 1.68 mg/mL, LC_{50} 2.17 mg/mL, LC_{50} 3.33 mg/mL	[13]

 https://doi.org/10.3390/plants14243720

Rosmarinus officinalis extract	*Rhipicephalus sanguineus*	LC_{50} 2.286 microlitre per ml; LC90 5.380 microlitre per ml	[14]
Ocimum gratissimum; *Ocimum canum*	Tick not specified	LC_{50} 1.01 % and LC_{50} 11.33 %; LC90 5.05 % and LC90 30.04 %	[15]
Ocimum gratissimum oil (December and September); p cymene; thymol; gamma terpinene	*Rhipicephalus microplus*	December LC_{50} 0.84 mg/mL; September LC_{50} 1.58 mg/mL; p cymene LC_{50} 1.41 mg/mL; thymol LC_{50} 1.81 mg/mL; gamma terpinene LC_{50} 3.08 mg/mL	[16]

Supplementary Table S2. GC–MS chromatographic analysis of plant extracts and essential oils used in ethnoveterinary tick control.

**Plant_or_extract**	**Key_compounds**	**Reference**
Rhododendron arboreum leaf extract	Limonene, Caryophyllene, 3 Heptanoic acid methyl ester, Phenol derivative	[17]
Momordica charantia ethanol extract	Vitamin E, Gentisic acid, Cucurbitacin B dihydro	[18]
Boswellia ovalifoliolata leaf extract	Secondary metabolites	[19]
Polyalthia longifolia, *Annona senegalensis*	Phthalates, Fatty acid esters, Caryophyllene	[20]
Dichrostachys cinerea fractions DCR1 and DCR2	Caryophyllene, Phthalate derivatives, Fatty acids	[21]
Sideritis sipylea essential oil and extract	Monoterpenes, Sesquiterpenes, Fatty acids	[22]
Osmanthus fragrans essential oil	Secoiridoids, Terpenoids, Phenolic glycosides	[23]
Telosma cordata extract	Primary and secondary metabolites	[24]
Euphorbia hirta, *Senna alata* extracts	Gamma tocopherol, Oleic acid, n Hexadecanoic acid, Quercetin, 9 Octadecenal	[25]
Malva sylvestris essential oil	Oleic acid, Palmitic acid, Phytone, p Vinylguaiacol, Phytol	[26]
Dittrichia graveolens extracts	Quinic acid glycosides, Gamma sitosterol, Sucrose	[27]
Artemisia herba alba, *Mentha pulegium*, *Cedrus atlantica* essential oils	Camphor, Pulegone, Beta himachalene	[28]
Citrus sinensis peel, leaf, flower oils	Limonene, Beta pinene, Terpenes	[29]
Scotch thistle extract (*Cirsium* species)	Arctiin, Arctigenin, Matairesinol	[30]
Gynochthodes ridsdalei stem extract	Sterols, Anthraquinones, Vitamins	[31]

Nardostachys chinensis essential oil	Terpenoids, Sesquiterpenes, Monoterpenes	[32]
Hyptis capitata extract	Diterpenes, Triterpenes, Sesquiterpenes, Esters, Alcohols	[33]
Rhododendron tomentosum essential oil	Myrica gale displays 1,8-cineole, alpha-terpineol, 4-terpineol, and thujenol; and Artemisia absinthium	[34]
Various essential oils	Terpenoid constituents	[35]
Beta vulgaris organic extracts	Fatty acid methyl esters, Organic acids	[36]
Citrus sinensis var balady peel oil	Beta pinene, Limonene, D Limonene	[37]
Multiple essential oil extracts	Terpenoids, Polyphenols, Saponins, Tannins, Flavonoids	[38]
Spilanthes paniculata extracts	Monoterpenes, Sesquiterpenes,	[39]
Calligonum comosum extracts	Volatile components	[40]
Prangos uloptera methanol extract	Butyl octanoate, 9 Octadecenoic acid, Alpha pinene	[41]
Citrus hystrix essential oil	Limonene, Citronellal, Linalool, Terpinen derivatives	[42]
Artemisia vulgaris essential oil	Terpenoid constituents	[43]
Rosmarinus officinalis essential oil	Verbenone, 1,8-cineol	[44]
Ocimum species essential oils	Limonene, Terpenoids, Sesquiterpenes	[45]
Capsicum frutescens methanol extract	Cis-13-octadecenoic acid	[46]
Essential oil extracts from multiple species	Furanosesquiterpenoids, Terpenoids	[47]
Various plant extracts	Terpenoids, Polyphenols, Aromatic esters	[48]

References

1. Alimi, D.; Hajri, A.; Jallouli, S.; Sebaï, H. Efficacy of Synergistic Activity of Seed Oils From Carthamus Tinctorius (Safflower) and Nasturtium Officinale (Watercress) on Lethality of the Cattle Tick Hyalomma Scupense (Acari: Ixodidae). *Open Veterinary Journal* **2022**, *12*, 80, doi:10.5455/ovj.2022.v12.i1.10.
2. Coşkun, Ş.; Girişgin, O.; Kürkçüoğlu, M.; Malyer, H.; Girişgin, A.O.; Kırımer, N.; Başer, K.H.C. Acaricidal Efficacy of Origanum Onites L. Essential Oil Against Rhipicephalus Turanicus (Ixodidae). *Parasitology Research* **2008**, *103*, 259-261, doi:10.1007/s00436-008-0956-x.
3. Godara, R.; Rafiqi, S.I.; Sharma, R.; Katoch, R.; Yadav, A.; Pilania, P.; Verma, P.K. Acaricidal Activity of Alstonia Scholaris and Sida Cordifolia Leaf Extracts Against Hyalomma Anatolicum Ticks. *The Indian Journal of Animal Sciences* **2021**, *90*, 1461-1464, doi:10.56093/ijans.v90i11.111487.
4. Coulibaly, A.; Hema, D.M.; Kiendrébéogo, M.; Nébié, R.C.H. Comparative Study of Two Monoterpenes Effect on Rhipicephalus Microplus Tick. *European Scientific Journal Esj* **2023**, *19*, 34, doi:10.19044/esj.2023.v19n24p34.
5. Castro, K.N.d.C.; Canuto, K.M.; Brito, E.S.d.; Costa, L.M.; Andrade, I.M.d.; Magalhães, J.A.; Barros, D.M.A. In Vitro Efficacy of Essential Oils With Different Concentrations of 1,8-Cineole Against Rhipicephalus

(Boophilus) Microplus. *Revista Brasileira De Parasitologia Veterinária* **2018**, *27*, 203-210, doi:10.1590/s1984-296120180015.

6. Koç, S.; Gültekin, Z.N.; Kahraman, Ş.; Cengiz, A.; Polat, B.; Calışkan, C.; Tufan-Çetin, Ö.; Çetin, H. Larvicidal and Repellent Effects of Essential Oils on the Brown Dog Tick (Rhipicephalus Sanguineus Sensu Lato) With Description of New Larval Repellent Activity Test Method. *Experimental and Applied Acarology* **2024**, *92*, 263-273, doi:10.1007/s10493-023-00892-2.
7. Nwanade, C.F.; Wang, M.; Wang, T.; Zhang, X.; Wang, C.; Yu, Z.; Liu, J. Acaricidal Activity of Cinnamomum Cassia (Chinese Cinnamon) Against the Tick Haemaphysalis Longicornis Is Linked to Its Content of (E)-Cinnamaldehyde. *Parasites & Vectors* **2021**, *14*, doi:10.1186/s13071-021-04830-2.
8. Lebouvier, N.; Hüe, T.; Hnawia, E.; Lesaffre, L.; Menut, C.; Nour, M. Acaricidal Activity of Essential Oils From Five Endemic Conifers of New Caledonia on the Cattle Tick Rhipicephalus (Boophilus) Microplus. *Parasitology Research* **2013**, *112*, 1379-1384, doi:10.1007/s00436-012-3268-0.
9. Taha, M.; Ali, A.A.B. Evaluation of Phytochemicals and Essential Oils of Cupressus Semprevirens in Controlling Cattle Tick Rhipicephalus Annulatus (Acari: Ixodidae). *BMC Plant Biology* **2025**, *25*, doi:10.1186/s12870-025-06222-5.
10. Araújo, L.X.; Novato, T.P.L.; Zeringóta, V.; Matos, R.d.S.; Senra, T.O.S.; Maturano, R.; Prata, M.C.A.; Daemon, E.; Monteiro, C.M.d.O. Acaricidal Activity of Thymol Against Larvae of Rhipicephalus Microplus (Acari: Ixodidae) Under Semi-Natural Conditions. *Parasitology Research* **2015**, *114*, 3271-3276, doi:10.1007/s00436-015-4547-3.
11. Figueiredo, A.; Nascimento, L.M.; Lopes, L.G.; Giglioti, R.; Albuquerque, R.D.D.G.d.; Santos, M.G.; Falcão, D.Q.; Nogueira, J.; Rocha, L.; Chagas, A.C.d.S. First Report of the Effect of Ocotea Elegans Essential Oil on Rhipicephalus (Boophilus) Microplus. *Veterinary Parasitology* **2018**, *252*, 131-136, doi:10.1016/j.vetpar.2018.02.018.
12. Castro, K.N.C.; Lima, D.F.; Vasconcelos, L.C.; Leite, J.R.; Santos, R.C.d.; Paz-Neto, A.A.; Costa, L.M. Acaricide Activity in Vitro of Acmella Oleracea Against Rhipicephalus Microplus. *Parasitology Research* **2014**, *113*, 3697-3701, doi:10.1007/s00436-014-4034-2.
13. Novato, T.P.L.; Araújo, L.X.; Monteiro, C.M.d.O.; Maturano, R.; Senra, T.d.O.S.; Matos, R.d.S.; Gomes, G.A.; Carvalho, M.G.d.; Daemon, E. Evaluation of the Combined Effect of Thymol, Carvacrol and (E)-Cinnamaldehyde on Amblyomma Sculptum (Acari: Ixodidae) and Dermacentor Nitens (Acari: Ixodidae) Larvae. *Veterinary Parasitology* **2015**, *212*, 331-335, doi:10.1016/j.vetpar.2015.08.021.
14. Abdelali, S.K.; Souttou, K.; Kacimi-Elhassani, M.; Aissaoui, L. Assessment of the Acaricidal Efficacy of Rosmarinus Officinalis Essential Oil Against Dogs' Ticks, Rhipicephalus Sanguineus (Acari: Ixodidae), and Its Chemical Composition. *Revista De Ciências Agroveterinárias* **2024**, *23*, 544-553, doi:10.5965/223811712332024544.
15. Kobenan, K.C.; Tia, V.E.; Ochou, G.E.C.; Kouakou, M.; Bini, K.K.N.; Dagnogo, M.; Dick, A.E.; Ochou, O.G. Comparaison Du Potentiel Insecticide Des Huiles Essentielles De Ocimum Gratissimum L. Et De Ocimum Canum Sims Sur Pectinophora Gossypiella Saunders (Lepidoptera : Gelechiidae), Insecte Ravageur Du Cotonnier en Côte D'Ivoire. *European Scientific Journal Esj* **2018**, *14*, 286, doi:10.19044/esj.2018.v14n21p286.
16. Lima, A.S.; Milhomem, M.N.; Monteiro, O.S.; Arruda, A.C.P.; Castro, J.A.M.; Fernandes, Y.M.L.; Maia, J.G.S.; Costa, L.M. Seasonal Analysis and Acaricidal Activity of the Thymol-Type Essential Oil of Ocimum Gratissimum and Its Major Constituents Against Rhipicephalus Microplus (Acari: Ixodidae). *Parasitology Research* **2017**, *117*, 59-65, doi:10.1007/s00436-017-5662-0.

17. Prakash, V. GC-MS (Gas Chromatography and Mass Spectroscopy) Analysis of Methanol Leaf Extract of Rhododendron Arboreum Sm. Of District Sirmaur, Himachal Pradesh. *Journal of Drug Delivery and Therapeutics* **2023**, *13*, 123-126, doi:10.22270/jddt.v13i1.5908.
18. Mohanty, S.K.; Nayak, Y.; Sahoo, L. Comprehensive Phytochemical Analysis of Momordica Charantia Ethanol Extract: Insights From Gas Chromatography-Mass Spectroscopy and in-Silico ADMET Studies. *Asian Journal of Agriculture* **2024**, *9*, doi:10.13057/asianjagric/g090101.
19. Venkateswarlu, G.; Singh, S.; Bodla, R. Phytochemical Evaluation, GC-MS Analysis of Ethanol Extract of Endemic Plant of ≪i>Boswellia Ovalifoliolata</I> Leaves. *Journal of Natural Remedies* **2025**, 215-222, doi:10.18311/jnr/2025/44410.
20. Shagal, M.H.; Baba, A.; Abdulkadir, Y.; Aisha, K.U. Isolation and Characterization of Methanolic Stem Bark Extracts of ≪i>Polyalthia Longifolia</I> And Root Extract of ≪i>Annona Senegalensis</I> Plants. *Journal of Health Wellness and Safety Research* **2025**, doi:10.70382/hujhwsr.v7i3.030.
21. Okwute, S.K.; Adeniyi, B.A. Phytochemical and Volatile Components Evaluation of Antimicrobial Root Extracts of ≪i>Dichrostachys Cinerea</I> (Sickle Bush)(Fabaceae</I>) (L) Wight &Amp; Arn. *Dutse Journal of Pure and Applied Sciences* **2024**, *10*, 316-324, doi:10.4314/dujopas.v10i3a.29.
22. Axiotis, E.; Petrakis, E.A.; Halabalaki, M.; Mitakou, S. Phytochemical Profile and Biological Activity of Endemic Sideritis Sipylea Boiss. In North Aegean Greek Islands. *Molecules* **2020**, *25*, 2022, doi:10.3390/molecules25092022.
23. Hu, C.; Liang, Y.Z.; Guo, F.-Q.; Li, X.; Wang, W. Determination of Essential Oil Composition From Osmanthus Fragrans Tea by GC-MS Combined With a Chemometric Resolution Method. *Molecules* **2010**, *15*, 3683-3693, doi:10.3390/molecules15053683.
24. Reme, S.; Keisar, L.D.; Chitra, R.; Ramalakshmi, A.; Kumaresan, D.; Manikanda, B.N. Phytochemical Profiling of Bioactive Compounds in Telosma Cordata (Tonkin Jasmine) Flower Extract Using GC-MS Technique. *Plant Science Today* **2025**, *12*, doi:10.14719/pst.11372.
25. Baba, R.T.; Oluboyo, B.O.; Egbebi, A.H.; Aladodo, M.; Dangana, Z.A.; Muhammad, Z.K.; Sowole, H.B.; Sani, H.U.; Yakubu, F. Investigation of the Phytochemical Constituents of ≪i>Euphorbia Hirta and Senna Alata</I> Using Qualitative, Quantitative and Gas Chromatography–Mass Spectrometry (GC-MS) Analysis. *Caliphate Journal of Science and Technology* **2025**, *7*, 282-290, doi:10.4314/cajost.v7i2.11.
26. Bekhradian, A.; Karami, B.; Rajabi, H.R. Green Synthesis of Silver/Silver Oxide Nanostructures Using the Malva Sylvestris Extract Prior to Simultaneous Distillation Extraction: Synthesis, Phytochemical and Biological Analysis. **2024**, doi:10.21203/rs.3.rs-4861630/v1.
27. Eltawaty, S.; Suliman, M.B.; El-Hddad, S.S.A.; Emgwer, H.; Shaieb, F. Phytochemical Screening, GC–MS Analysis, and Antibacterial Activity of Dittrichia Graveolens (L.) Greuter. *Tropical Journal of Natural Product Research* **2025**, *9*, 1476, doi:10.26538/tjnpr/v9i4.14.
28. Oualdi, I.; Merzouki, M.; Ouahhoud, S.; Chakrone, K.; Benabbes, R.; Yousfi, E.B.; Challioui, A.; Hammouti, B.; Touzani, R. Essential Oils of Artemisia Herba-Alba, Mentha Pulegium, and Cedrus Atlantica: Chemical Compositions, in Vitro, in Vivo, in Silico Antifungals Activities, and Genotoxicity. *Asean Journal of Science and Engineering* **2025**, *5*, 45-60, doi:10.17509/ajse.v5i1.80693.
29. Bhandari, D.P.; Chaudhary, P.; Upadhyaya, S.R.; Ranjitkar, R.; Satyal, R.; Adhikari, A.; Satyal, P.; Parajuli, N. Chemical Variability, Antioxidant and Larvicidal Efficacy of EOs From Citrus Sinensis (L.) Osbeck Peel, Leaf, and Flower. *Horticulturae* **2024**, *10*, 566, doi:10.3390/horticulturae10060566.

30. Mohammadi, S.; Movefeghi, A.; Delazar, A.; Hamedeyazdan, S.; Bahadori, M.B.; Nazemiyeh, H. <b>Isolation and Characterization of Bioactive Compounds From Scotch Thistle (<i>Onopordum Acanthium</I> L.) Seeds</B>. *Pharmaceutical Sciences* **2025**, *31*, 288-293, doi:10.34172/ps.025.40963.
31. Nair, R.R.; Gangaprasad, A. Gc-MS Analysis of Methanolic Stem Extract of Gynochthodes Ridsdalei, Razafim and B. Bremer, an Endemic, Endangered Medicinal Plant of Southern Western Ghats. *International Journal of Current Pharmaceutical Research* **2017**, *9*, 98, doi:10.22159/ijcpr.2017.v9i3.19665.
32. Wang, F.; Liu, S.; Luo, M.; Qin, Y.; Pan, L.; Liu, Y.; Yan, L. Analysis of Essential Oil Of<i>Nardostachys Chinensis</I>Batal by GC-MS Combined With Chemometric Techniques. *Acta Chromatographica* **2015**, *27*, 157-175, doi:10.1556/achrom.27.2015.1.12.
33. Susanti, Y.; A'yun, A.Q. Phytochemical, Antioxidant, and Antibacterial Activity of Essential Oil Hyptis Capitata Using Solvent-Free Microwave Extraction. *Journal of Applied Agricultural Science and Technology* **2024**, *8*, 450-460, doi:10.55043/jaast.v8i4.275.
34. Jaenson, T.G.T.; Pålsson, K.; Borg-Karlson, A.K. Evaluation of Extracts and Oils of Tick-repellent Plants From Sweden. *Medical and Veterinary Entomology* **2005**, *19*, 345-352, doi:10.1111/j.1365-2915.2005.00578.x.
35. Kyasa, S.K. Investigating Terpenoid Constituents in Commercial Essential Oils to Learn and Practice GC–MS and Literature Research Skills. *Journal of Chemical Education* **2020**, *97*, 1966-1969, doi:10.1021/acs.jchemed.0c00046.
36. Fiadorwu, J.; Subedi, K.; Todd, D.A.; Basti, M.M. Multipronged Approach to Profiling Metabolites in ≪em>Beta Vulgaris</Em> L Dried Pulp Extracts Using Chromatography, NMR, and Other Spectroscopy Methods. **2023**, doi:10.20944/preprints202308.0182.v1.
37. Ashour, M.D.A.M.S.H.; Hafez, S.; Habeeb, S.M.; Allam, N.A.T. Proteomics and Metabolic Patterns of Hyalomma Dromedarii Ticks Treated With Citrus Sinensis Var Balady Peels' Oil Extract. *Egyptian Journal of Veterinary Sciences* **2023**, *54*, 1073-1095, doi:10.21608/ejvs.2023.206959.1493.
38. Ugwu, C.N.; Ezinwanne, O.B.; Victor, O.N.; Attama, A.A. Evaluation of Antibacterial Properties and Gas Chromatography-Mass Spectroscopy (Gc-Ms) Profile of Essential Oil From Lemon Peel. *African Journal of Pharmaceutical Research and Development* **2025**, *17*, 96-113, doi:10.59493/ajopred/2025.1.11.
39. Satapathy, S.S.; Bhuyan, R.; Sahoo, A.; Sahoo, C.; Bhuyan, S.K. Phytochemical Profiling of All Parts of Spilanthes Paniculata (Toothache Plant) for Therapeutic Implications. *Research Journal of Biotechnology* **2024**, *19*, 79-84, doi:10.25303/1912rjbt079084.
40. Masoum, S.; Seifi, H.; Ebrahimabadi, E.H. Characterization of Volatile Components in Calligonum Comosum by Coupling Gas Chromatography-Mass Spectrometry and Mean Field Approach Independent Component Analysis. *Analytical Methods* **2013**, *5*, 4639, doi:10.1039/c3ay40451j.
41. Valibeik, A.; Tavakoli, N.; Amiri, H.; Heydari, R.; Hasanvand, L.; Rostami, R.; Dastjerd, N.T.; Ahmadvand, H. Composition of the Essential Oils, Antioxidant and Antibacterial Activities of the Methanolic Extract of <i>Prangos Uloptera</I>. *Immunopathologia Persa* **2022**, doi:10.34172/ipp.2022.29278.
42. Tran, T.H.; Quyen, N.T.C.; Trúc, T.T.; Quân, P.M. Evaluate the Chemical Composition of Kaffir Lime (Citrus Hystrix) Essential Oil Using the Classical Method. *Iop Conference Series Materials Science and Engineering* **2020**, *991*, 012014, doi:10.1088/1757-899x/991/1/012014.
43. Sapkota, S.; Kadariya, I.P.; Pandey, M.; Risal, P.; Basnet, B.B. Antioxidant Activity of Essential Oil of Artemisia Vulgaris Collected From Sub-Tropical Region of Bagmati Province, Nepal. *Journal of Agriculture and Forestry University* **2022**, 203-207, doi:10.3126/jafu.v5i1.48466.

44. Al-Hayali, O.Z.; Marjani, M.F.A.L.; Maleki, A. Evaluation of Rosmarinus Officinalis Leaves Essential Oils Activity Against Vancomycin Intermediate Staphylococcus Aureus (VISA) Isolated From Baghdad Hospital Patients. *Iraqi Journal of Science* **2023**, 2153-2167, doi:10.24996/ijs.2023.64.5.5.
45. Hüe, T.; Cauquil, L.; Fokou, J.B.H.; Dongmo, P.M.J.; Bakarnga-Via, I.; Menut, C. Acaricidal Activity of Five Essential Oils of Ocimum Species on Rhipicephalus (Boophilus) Microplus Larvae. *Parasitology Research* **2014**, *114*, 91-99, doi:10.1007/s00436-014-4164-6.
46. Kishore, V.; Loach, N.; Srivastava, C.N.; Mohan, L. Toxicity Evaluation and Chemical Composition of Capsicum Frutescens for Natural Control of Asian Blue Tick, Rhipicephalus (Boophilus) Microplus (Acari: Ixodidae). *The Journal of Basic and Applied Zoology* **2021**, *82*, doi:10.1186/s41936-021-00249-4.
47. Maggi, F.; Papa, F.; Giuliani, C.; Bini, L.; Venditti, A.; Bianco, A.; Nicoletti, M.; Iannarelli, R.; Caprioli, G.; Sagratini, G.; et al. Essential Oil Chemotypification and Secretory Structures of the Neglected Vegetable <i>Smyrnium Olusatrum</I> L. (Apiaceae) Growing in Central Italy. *Flavour and Fragrance Journal* **2014**, *30*, 139-159, doi:10.1002/ffj.3221.
48. Pazinato, R.; Volpato, A.; Baldissera, M.D.; Santos, R.C.V.; Baretta, D.; Vaucher, R.d.A.; Giongo, J.L.; Boligon, A.A.; Stefani, L.M.; Silva, A.S.D. In Vitro Effect of Seven Essential Oils on the Reproduction of the Cattle Tick Rhipicephalus Microplus. *Journal of Advanced Research* **2016**, *7*, 1029-1034, doi:10.1016/j.jare.2016.05.003.
